# Impact of sepsis on the dynamics of diaphragmatic function in patients under mechanical ventilation

**DOI:** 10.1186/2197-425X-3-S1-A94

**Published:** 2015-10-01

**Authors:** M Dres, BP Dubé, L Dangers, J Mayaux, T Similowski, A Demoule

**Affiliations:** Hopital Pitie Salpetriere, Assistance Publique Hôpitaux de Paris, Respiratory and Critical Care Department, Paris, France; UPMC Université Paris 06, INSERM, UMRS1158 Neurophysiologie Respiratoire Expérimentale et Clinique, Sorbonne Universités, Paris, France

## Introduction

Sepsis and mechanical ventilation are both risk factors of diaphragmatic dysfunction (DD) in critically ill patients. The evolution of diaphragmatic function under mechanical ventilation in septic and non-septic patients has not been described.

## Objectives

To assess the evolution of diaphragmatic function in patients under mechanical ventilation (MV) with and without sepsis on admission.

## Methods

We prospectively included mechanically ventilated patients of a 16-beds medical intensive care unit. Diaphragmatic function was estimated by the gold standard method using bilateral anterior magnetic stimulation (Ptr,stim) at two distinct time points:

1) during the first 24 hours of mechanical ventilation and

2) in the 24 hours after switching to spontaneous ventilation mode. Sepsis was defined according to the Surviving Sepsis Campaign criteria. A Ptr,stim lower than -11 cmH_2_O defined DD.

## Results

Forty-two critically ill patients were investigated (26 males, mean age 61 ± 14 years, SOFA score 9 ± 4). Twenty six (62%) had sepsis on admission. Patients with and without sepsis were similar with regards to age, gender, past medical conditions, length of stay and of MV, use of sedatives and SOFA score). The first and second evaluations were respectively performed after a median (interquartile range) of 1(1-1) and 3.75 (2-5) days from the onset of MV. Overall, DD was present in 34 patients (81%) on the first evaluation and in 35 (83%) on the second. Initial Ptr,stim was lower in patients with sepsis (6.6 ± 3.2 vs 9.7 ± 4.8 cmH_2_O, p = 0.016). Patients with initial sepsis had a mean increase of 29.7% in Ptr,stim between the two evaluations, whereas in patients without initial sepsis Ptr,stim decreased by a mean 10.9% (p = 0.03). On average, Ptr,stim increased by 12.4 percent.day^-1^ and decreased by 3.8 percent.day^-1^ in septic and non septic patients, respectively.

## Conclusions

In critically ill patients, sepsis on admission is associated with worse but partially reversible diaphragmatic dysfunction. On the opposite, patients without sepsis had a continuous decrease in diaphragm function. These findings provide new insight in the interrelated contributions of the sepsis and MV on diaphragmatic dysfunction.

## Grant Acknowledgment

Martin DRES was supported by Assistance Publique Hôpitaux de Paris

